# Correlation of Positive Psychological Health among US Adults (Aged ≥ 50 Years) with Pain and Documented Opioid Treatment

**DOI:** 10.3390/bs14010027

**Published:** 2023-12-29

**Authors:** David R. Axon, Uche Agu

**Affiliations:** 1 Department of Pharmacy Practice and Science, College of Pharmacy, The University of Arizona, 1295 North Martin Avenue, P.O. Box 210202, Tucson, AZ 85721, USA; usagu@arizona.edu; 2 Center for Health Outcomes & Pharmacoeconomic Research (HOPE Center), College of Pharmacy, The University of Arizona, 1295 North Martin Avenue, P.O. Box 210202, Tucson, AZ 85721, USA

**Keywords:** psychological health, pain, older adults, opioid, cross-sectional studies

## Abstract

In this study, we aimed to identify the factors correlated with positive psychological health among United States older adults (≥50 years) with pain and documented opioid treatment. This retrospective cross-sectional study utilized a nationally representative dataset (Medical Expenditure Panel Survey). A multivariable logistic regression model was developed to assess the correlation of positive psychological health in the eligible population. The logistic regression model showed having excellent/very good/good (versus fair/poor) perceived health (adjusted odds ratio [AOR] = 9.062; 95% confidence interval [CI] = 5.383, 15.254) had a statistically significant correlation with positive psychological health among the eligible population. This finding offers important insights for clinicians and policymakers to consider when formulating approaches to better manage the psychological health of United States older adults with pain and documented opioid treatment.

## 1. Introduction

Pain has been defined as a distressing sensory and emotional encounter linked to or similar to that related to genuine or potential harm to bodily tissues [1]. This definition further suggests that pain, which is a subjective encounter, could be affected to different extents by psychological, social, and biological factors. Therefore, pain could serve as an important precursor to underlying health conditions as well as an indicator for disease diagnosis [2].

According to the Centers for Disease Control and Prevention (CDC), about 20.4% of older people in the United States (US) suffer from pain-related ailments, and this figure tends to increase with age [3]. Additionally, after adjusting for age, females, people living near or below the poverty line, and people living in rural areas have been shown to have higher odds of pain [3].

Research focused on pain-related healthcare utilization reveals that pain contributes to a substantial reason for individuals to seek medical assistance [4]. Furthermore, neck pain, musculoskeletal disorders, and back pain are three of the primary reasons for years lost to disability [5].

Similarly, pain incurs significant economic costs, as patients with pain conditions use considerably more medical resources than others [6]. According to a 2010 report from the Institute of Medicine, about 33% of people in the US suffer from chronic pain, with costs ranging from USD 560 to 635 billion per annum in medical cost and productivity losses, respectively [7]. Additionally, studies show that the yearly expense of long-term pain surpasses chronic conditions such as cardiovascular disease and cancer [8]. These figures did not factor in the expenses associated with caring for incarcerated individuals, nursing home residents, military personnel, children, and caregiving [9].

The economic and healthcare burden of pain has evidently contributed to poor quality of life of pain sufferers [10,11]. For instance, pain has been associated with limitations in daily or physical activities [10,11], opioid addiction, anxiety, and depression [10]. Also, pain has been shown to negatively influence relationships and self-esteem, linked to higher divorce and suicide rates [12,13,14], and is associated with reduced life expectancy [15].

In terms of chronic pain management, prescription drugs such as opioids and non-steroid anti-inflammatory drugs are often used [16,17]. However, guidelines no longer recommend opioids as the early option for any form of long-term pain, particularly for younger persons or pain not related to cancer [18]. This is because opioids have been linked to increased rates of addiction, overdose mortality, or increased hospitalization [19].

Considerable overlap exists between pain and psychological health [20], and it is widely recognized that pain and psychiatric diagnoses are frequently comorbid [21,22,23]. It has been suggested that this link is likely a two-way relationship [24,25], as individuals may develop depression or anxiety due to pain and vice versa [26,27]. In psychiatric settings, physical pain symptoms are reported by 50% of patients with depression [28], and a psychiatric diagnosis increases the odds of opioid misuse [29,30,31]. This finding is supported by one of the earliest studies into positive psychological health. This study showed that positive health behavior is associated with a sense of psychological well-being and is more likely to be prevalent among certain demographics [32]. A more recent study into the factors predictive of positive psychological health corroborates the role of positive health behavior as an important contributor to positive psychological health [33]. Similarly, another recent study suggests that healthy living such as physical activity and avoidance of illicit substances can improve psychological well-being [34]. Although advancing age is associated with physical debility, it is worth noting that some older adults exhibit low anxiety and increased happiness with advancing age [35].

Therefore, it is crucial to understand the correlation between the characteristics of older pain patients and psychological health, given the higher odds of psychological health issues and the potential negative effects of opioid use in this population [36]. To address this issue, we utilized a dataset from the Medical Expenditure Panel Survey (MEPS), which represents the US population, to identify characteristics correlated with psychological health in this population.

## 2. Materials and Methods

### 2.1. MEPS Dataset

MEPS, which is a US-specific dataset, is a comprehensive collection of surveys that has been in operation since 1996. It was designed to gather information on families, individuals, healthcare providers, and employers in the country. Through these surveys, MEPS gathers data on variables such as the types of healthcare services used by people, the frequency of use, associated costs, payment modes, and the number and costs of health insurance coverage available to people. By utilizing suitable weighting factors, MEPS has the added benefit of generating estimates of the US population that are representative at the national level for individuals who are not living in institutions.

The Household Component of the MEPS dataset is a rich resource of information related to participants’ health status, socioeconomic and demographic characteristics, employment status, healthcare access, and level of satisfaction with health services. Based on this information, it is possible to create estimates for individuals, families, and specific population subgroups. The survey has a panel design that comprises five sets of interviews spanning two full calendar years. In 2020, due to COVID-19’s impact on the number of completed interviews, two additional rounds of interviews were included. These data can be leveraged to track changes in specific variables at the individual level, such as health costs, health insurance, and health status.

For our study, we utilized the full year consolidated and the prescribed medicines files of the 2020 MEPS dataset (which were the most recent files existing at the initiation of our research). The data contained in the Prescribed Medicines data file present comprehensive details about the prescribed medicines reported by households in a sample that represents the US civilian noninstitutionalized population. This information can be utilized to calculate the annual estimates of the expenses and utilization of prescribed medicines [37,38,39].

### 2.2. Eligibility Criteria

Study subjects were included in our sample for analysis if they had all the following characteristics: alive all through the target year (2020), 50 years of age or above, experienced pain symptoms within the past four weeks, and had an opioid (or combination opioid) prescription within the target year.

The presence of pain was established by asking respondents to interpret the extent to which it affected their normal work outside the home and housework in the past four weeks. Response options included “a little bit”, “moderately”, “quite a bit”, and “extremely”. Respondents who received an opioid medication were detected using the codes 60 (opioid analgesics) and/or 191 (combination of opioid analgesics) from the data file [38,39].

### 2.3. Outcome Variable

The outcome in our analysis was psychological health, which was classified as positive or negative. The classifications were formulated depending on answers to the survey items which requested subjects to categorize their psychological health as excellent, very good, good, fair, or poor. For our analysis, we modified responses of ‘excellent, very good, and good’ to ‘positive psychological health’, while responses of ‘fair or poor’ were modified to ‘negative psychological health’.

### 2.4. Independent Variable

The independent variables in this study were selected based on the presence of at least some evidence that they influence psychological health. These variables included patient characteristics such as age in years [40]; sex [41]; race [42]; ethnicity [43]; marital status [44]; education completed [45]; employment status [46]; income level [47]; insurance coverage [48]; census region [49]; chronic conditions [50]; perceived health [51]; exercise [52]; smoker [53]; body mass index [54]; activity restrictions [55]; and pain severity [56].

### 2.5. Data Analysis

The descriptive characteristics of study subjects in both groups (positive vs negative psychological health) were compared via chi-square tests. A logistic regression model was developed to assess statistically significant correlations between variables and positive psychological health, with negative psychological health serving as the reference group. This was reported using the adjusted odds ratio and a 95% confidence interval. The a priori alpha level was set at 0.05. Only variables that had a *p*-value < 0.05 in the descriptive analysis were included in the logistic regression analysis (i.e., education completed, employment status, income level, insurance coverage, perceived health, exercise, smoker, activity restrictions, and pain severity). The MEPS complex survey design was accounted for using cluster, stratum, and weighting variables to obtain nationally generalizable estimates. A domain analysis was conducted to differentiate the eligible population from the ineligible population. Collinearity was assessed with a correlation matrix where values ≥ 0.8 indicated collinearity. Missing data were not included in the analysis. All analyses were done using SAS University Edition (SAS institute Inc., Cary, NC, USA).

## 3. Results

**Figure 1:**
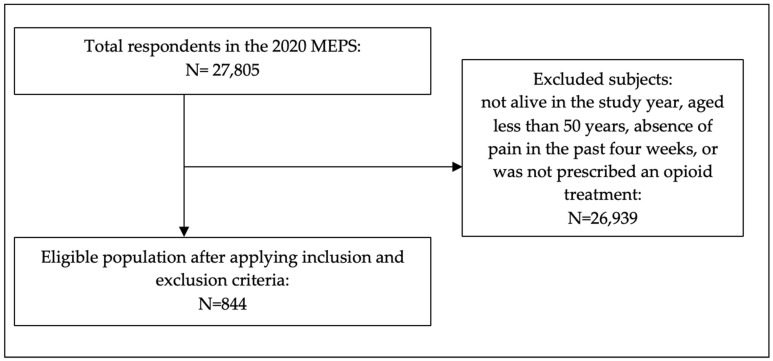
Study subject eligibility.

In this study, 844 participants were involved, out of which 668 considered their psychological health to be positive, while 176 reported perceiving their psychological health as negative. After applying the survey weights, this was converted to a population of 10,602,045 participants, of which 80% (95% confidence interval (CI) = 76.7%, 83.4%) considered their psychological health to be positive, while 20% (95% CI = 16.6%, 23.3%) reported perceiving their psychological health as negative. See Figure 1.

The characteristics of US adults (age ≥ 50 years) with pain and documented opioid treatment, stratified by positive and negative psychological health, are shown in Table 1. Notable variations, which were statistically significant, among positive and negative psychological health groups were observed in certain characteristics including education completed (*p* = 0.0454), employment status (*p* < 0.0001), income level (*p* = 0.0002), insurance coverage (*p* = 0.0044), perceived health (*p* < 0.0001), exercise (*p* < 0.0001), smoking (*p* = 0.0014), activity restriction (*p* < 0.0001), and pain severity (*p* < 0.0001).

Table 2 shows the correlation of positive (versus negative) psychological health status among United States older adults (age ≥ 50 years) with pain and documented opioid treatment. Based on the logistic regression analysis, it was found that the only factor that correlated with positive psychological health was excellent/very good/good perceived health (adjusted odds ratio (AOR) = 9.062; 95% CI = 5.383, 15.254). There was no evidence of collinearity in the logistic regression model (no correlations ≥ 0.8). The likelihood ratio value, score value, and Wald value were all <0.0001, and the model c-statistic was 0.808.

## 4. Discussion

Our study found that perceived health status was significantly correlated with psychological health in the eligible population. The finding that perceived health was correlated with psychological health seems reasonable as it agrees with results from similar studies. For instance, Axon et al., in a recent study using MEPS data, found that self-rated physical health status was a significant predictor of emotional wellness in older pain patients [57]. Similarly, a study by Amstadter et al. found a link between negative health status in older adults and psychological wellness [58]. Another study by Ohrnberger et al. showed that past physical health had an influence on mental wellness, largely through physical activity. Additionally, their research showed that past physical health had a greater impact on present psychological health than economic status or education level [59,60]. These findings support the increased consideration of the physical health needs of older patients with pain, especially those using opioid medications.

Interestingly, none of the other patient characteristics in this study were found to be correlated with psychological health. This differs from the findings from similar studies that found that factors such as employment [46], education status [45], income level [47], health insurance [48], exercise [52], smoking status [53], activity limitations [55], and pain severity [56] were associated with psychological health. The finding that employment status was not correlated was more interesting considering the available evidence [61,62,63,64,65]. For instance, studies have shown that elderly individuals are more susceptible to incurring higher medical costs or becoming financially dependent on others when they retire from work [66]. These financial challenges could lead to depression [67]. Therefore, future studies should investigate the economic situation of older adults who experience both pain and deteriorating psychological health. This is because meaningful employment could contribute to improved mental health by providing avenues for social interaction, mental stimulation, and a sense of purpose [68,69,70]. Furthermore, a study by Axon et al. that used 2017 MEPS data found that education level and activity limitation were associated with psychological health [57]. These differences could be explained by the fact that our study used narrowly defined eligibility criteria to improve the precision of our target population. For instance, our study was focused on a subgroup of older adults (age ≥50 years) with pain who recently used an opioid. In addition, we used a more recent dataset that included a population that was alive all through the target year, experienced pain symptoms within the past four weeks, and had an opioid (or combination opioid) prescription within the target year.

Findings from this study demonstrate potential value in the investigational and treatment approaches utilized in improving the quality of life for this category of patients. Considering that many studies have demonstrated correlations between pain and psychological health [20], as well as the role of opioids in the deterioration of psychological health [29,30,31,71], the results of this analysis serve to provide healthcare providers and policy makers with factors to consider when managing the psychological health of older patients with pain who also use an opioid medication. Studies have also shown that elderly patients are at a higher risk of psychological health deterioration [36]. Hence, understanding the potential factors that are correlated with the psychological health of these patients is an important clinical consideration. Since it has been established that higher healthcare expenditures are expected among older opioid medication users experiencing pain [72], this study will serve to inform key economic considerations in the management of this group of patients. Furthermore, while past studies have evaluated the connection between pain and psychological health or between opioid use and psychological health [20,29,30,31,71], our study adds a fresh perspective to the literature by using the MEPS dataset, which is more representative of the US population.

Our study has some limitations. First, there is a tendency for recall bias since our research is derived from data pooled from a cross-sectional survey. However, the frequent MEPS data collection, which occurs every 4 to 5 months, helps to minimize such a limitation. Second, our study was not able to differentiate between types of pain given that only one definition was available in the MEPS dataset, and other definitions exist. It is important to note that pain is a subjective condition, and that exposure to pain (and perception of pain) can vary from person to person. Furthermore, our study was not able to differentiate between acute and chronic use of opioids since opioid use was determined based on any prescription within the calendar year. We could also not confirm whether opioids were used at the same time the person had pain during this one-year timeframe. Finally, although the study design did not establish a causal relationship, it did reveal a statistical correlation between psychological health and two variables (perceived health and employment status). To build on these findings, future research could explore whether interventions targeting the two factors associated with psychological health could lead to changes in psychological health among older adults who were prescribed opioids. Future research could also involve a similar study comparing psychological health among US adults with pain who used opioids versus those who did not use opioids, which may speak to the impact of opioid use on psychological health.

## 5. Conclusions

To summarize, this is the first study that utilized the MEPS dataset, which represents the US population, to evaluate variables correlated with psychological health among older adults experiencing symptoms of pain and having recently used an opioid medication. A statistically significant factor (perceived health) was found to be correlated with psychological health among our target population. This finding offers important insights for clinicians and policymakers to consider when formulating approaches to better manage the psychological health status of older patients with pain who have recently used an opioid medication. This finding could also help to emphasize the need and inform the shift from secondary prevention to predictive medicine for psychological health in this population. Opportunities exist for future studies to assess causality and appraise the effect of any interventions utilized to improve psychological health in this population.

## Figures and Tables

**Table 1:** Characteristics of US adults (age ≥ 50 years) with pain and documented opioid treatment, stratified by positive and negative psychological health.

Variables	Positive Psychological Health N = 668 (Weighted N = 8,482,566) Weighted % (95% Confidence Interval)	Negative Psychological Health N = 176 (Weighted N = 2,119,480) Weighted % (95% Confidence Interval)	*p*
Age (years)			0.2327
50–64	45.8 (40.7, 50.8)	52.4 (42.6, 62.1)	
≥65	54.2 (49.2, 59.3)	47.6 (37.9, 57.4)	
Sex			0.4830
Male	38.7 (34.4, 43.0)	42.5 (32.7, 52.2)	
Female	61.3 (57.0, 65.6)	57.5 (47.8, 67.3)	
Race			0.6719
White	82.6 (79.2, 86.0)	80.9 (73.9, 88.0)	
Another	17.4 (14.0, 20.8)	19.1 (12.0, 26.1)	
Ethnicity			0.4259
Hispanic	5.8 (3.6, 7.9)	7.5 (3.1, 12.0)	
Non-Hispanic	94.2 (92.1, 96.4)	92.5 (88.0, 96.9)	
Marital status			0.0819
Married	52.1 (47.6, 56.6)	42.1 (32.3, 52.0)	
Other	47.9 (43.4, 52.4)	57.9 (48.0, 67.7)	
Education completed			0.0454
High school or less	46.0 (42.1, 49.9)	57.2 (46.7, 67.6)	
More than high school	54.0 (50.1, 57.9)	42.8 (32.4, 53.3)	
Employment status			<0.0001
Employed	32.9 (28.5, 37.4)	9.4 (4.2, 14.7)	
Unemployed	67.1 (62.6, 71.5)	90.6 (85.3, 95.8)	
Income level			0.0002
Poor/near poor/low income	33.2 (28.7, 37.6)	53.5 (43.4, 63.5)	
Middle/high income	66.8 (62.4, 71.3)	46.5 (36.5, 56.6)	
Insurance coverage			0.0044
Private	52.67 (48.2, 57.2)	35.8 (25.3, 46.3)	
Public	47.0 (42.5, 51.4)	63.6 (53.4, 73.9)	
Uninsured	0.3 (0.0, 0.7)	0.6 (0.0, 1.7)	
Census region			0.8325
Northeast	14.4 (10.3, 18.5)	15.9 (8.9, 23.0)	
Midwest	23.1 (19.1, 27.1)	24.7 (17.4, 32.1)	
South	44.4 (39.2, 49.6)	44.6 (35.0, 54.2)	
West	18.1 (14.3, 21.9)	14.8 (8.7, 20.8)	
Chronic conditions			0.0878
<2	12.4 (9.4, 15.5)	5.2 (0.0, 10.5)	
≥2	87.6 (84.5, 90.6)	94.8 (89.5, 100.0)	
Perceived health			<0.0001
Excellent/very good/good	71.4 (67.0, 75.8)	15.4 (8.9, 21.9)	
Fair/poor	28.6 (24.2, 33.0)	84.6 (78.1, 91.1)	
Exercise			<0.0001
Yes	39.9 (35.2, 44.6)	19.0 (12.0, 26.1)	
No	60.1 (55.4, 64.8)	81.0 (73.9, 88.0)	
Smoker			0.0014
Yes	12.9 (10.1, 15.7)	24.9 (17.0, 32.8)	
No	87.1 (84.3, 89.9)	76.1 (67.2, 83.0)	
Body Mass Index			0.2464
Overweight/obese	74.9 (70.5, 79.4)	80.3 (72.7, 87.8)	
Normal/underweight	25.1 (20.6, 29.5)	19.7 (12.2, 27.3)	
Activity restrictions			<0.0001
Yes	66.1 (61.5, 70.7)	91.4 (86.5, 96.3)	
No	33.9 (29.3, 38.5)	8.6 (3.7, 13.5)	
Pain severity			<0.0001
Little/moderate	61.4 (57.1, 65.7)	26.3 (17.4, 35.2)	
Quite a bit/extreme	38.6 (34.3, 42.9)	73.7 (64.8, 82.6)	

A total of 844 (unweighted) US adults (≥50 years) who had pain and documented opioid treatment were included in the analysis. The differences between these groups were evaluated using chi-square tests.

**Table 2:** Correlation of positive (versus negative) psychological health status among United States older adults (age ≥ 50 years) with pain and documented opioid treatment.

Factors	Adjusted Odds Ratio (95% Confidence Limits)
High school or less vs. higher than high school education	1.092 (0.679, 1.756)
Employed vs. unemployed	2.048 (0.986, 4.255)
Poor/near poor/low income vs. middle/high income	0.623 (0.379, 1.022)
Private vs. uninsured insurance coverage	1.381 (0.195, 9.757)
Public vs. uninsured insurance coverage	1.908 (0.294, 12.391)
Excellent/very good/good vs. fair/poor perceived health	**9.062** (**5.383, 15.254**)
Exercise yes vs. no	1.411 (0.804, 2.475)
Smoker yes vs. no	0.807 (0.434, 1.502)
Activity restrictions yes vs. no	0.616 (0.289, 1.323)
Little/moderate vs. quite a bit/extreme pain severity	1.785 (1.000, 3.188)

Analysis based on a sample of 844 (unweighted) US adults living in 2020, age ≥ 50 years, with pain and documented opioid treatment. Bold indicates that the variable has a significant correlation with positive psychological health.

## Data Availability

Data are available from the corresponding author upon reasonable request.
